# The effectiveness of a web-based brief alcohol intervention in reducing heavy drinking among adolescents aged 15 to 20 years with a low educational background: study protocol for a randomized controlled trial

**DOI:** 10.1186/1745-6215-13-83

**Published:** 2012-06-15

**Authors:** Carmen V Voogt, Evelien A P Poelen, Lex A C J Lemmers, Rutger C M E Engels

**Affiliations:** 1 Behavioural Science Institute, Radboud University Nijmegen, Montessorilaan 3, 6525 HR, Nijmegen, The Netherlands; 2 Trimbos Institute, Netherlands Institute of Mental Health and Addiction, Da Costakade 45, 3500 AS, Utrecht, The Netherlands

**Keywords:** Heavy drinking, adolescents with a low educational background, web-based brief alcohol intervention

## Abstract

**Background:**

The serious negative health consequences of heavy drinking among adolescents is cause for concern, especially among adolescents aged 15 to 20 years with a low educational background. In the Netherlands, there is a lack of alcohol prevention programs directed to the drinking patterns of this specific target group. The study described in this protocol will test the effectiveness of a web-based brief alcohol intervention that aims to reduce alcohol use among heavy drinking adolescents aged 15 to 20 years with a low educational background.

**Methods/design:**

The effectiveness of the What Do You Drink (WDYD) web-based brief alcohol intervention will be tested among 750 low-educated, heavy drinking adolescents. It will use a two-arm parallel group cluster randomized controlled trial. Classes of adolescents from educational institutions will be randomly assigned to either the experimental (n = 375: web-based brief alcohol intervention) or control condition (n = 375: no intervention). Primary outcomes measures will be: 1) the percentage of participants who drink within the normative limits of the Dutch National Health Council for low-risk drinking, 2) reductions in mean weekly alcohol consumption, and 3) frequency of binge drinking. The secondary outcome measures include the alcohol-related cognitions, attitudes, self-efficacy, and subjective norms, which will be measured at baseline and at one and six months after the intervention.

**Discussion:**

This study protocol presents the study design of a two-arm parallel-group randomized controlled trial to evaluate the effectiveness of the WDYD web-based brief alcohol intervention. We hypothesized a reduction in mean weekly alcohol consumption and in the frequency of binge drinking in the experimental condition, resulting from the web-based brief alcohol intervention, compared to the control condition.

**Trial registration:**

Netherlands Trial Register NTR2971

## Background

Heavy alcohol use among adolescents continues to be a great public health concern in most Western countries, given the immediate and long-term health consequences [1]. In the Netherlands, the prevalence of heavy drinking is particularly high among adolescents with a low educational background, aged 15 to 20 years [2-4].

Adolescents with a low educational background not only engage in heavy drinking more often, but also start drinking at a younger age compared to higher educated peers [4-6]. Possible explanations for this difference are that they spend more time with friends, are raised more often in single-parent families, experience less rule setting and monitoring by the mother, and engage more often in externalizing behaviors [3].

Both early drinking onset and heavy drinking can place low-educated adolescents at an increased risk for developing acute and long-term health consequences, such as alcohol-related violence [7], drunk driving, injuries and risky sexual behavior [8]. This behavior is also associated with brain impairment and neurocognitive deficits, which have implications for learning and intellectual development [9,10]. In the long term, heavy drinking is predictive of, among other things, problematic adult alcohol use [11], liver cirrhosis [12], specific types of cancer, and cardiovascular disease [1]. From a public health viewpoint, it is crucial to develop alcohol prevention programs directed at adolescents with lower education levels to encourage them to change their risky drinking practices, especially considering that 60% of all adolescents, following secondary education in the Netherlands, are low-educated [3]. The study described in this protocol will test the effectiveness of the WDYD web-based brief alcohol intervention that aims to reduce alcohol use among heavy-drinking adolescents aged 15 to 20 years with a low educational background.

The school system in the Netherlands comprises several types of education. After eight years of primary education, pupils go directly to secondary education, which consists of preparatory secondary vocational education (VMBO), senior general education (HAVO), and preuniversity education (VWO). Pupils with a VMBO diploma are able to attend a secondary vocational education (MBO), which has four learning routes: 1) the theoretical route allowing admission to MBO or HAVO, 2) a mixed educational route, 3) the vocationally oriented route, and 4) a vocational route allowing pupils to enter the labor market directly. HAVO prepares pupils for higher professional education (HBO), while VWO prepares pupils for university.

In the Netherlands, there is a lack of evidence-based alcohol prevention programs targeting adolescents following the lower education levels [13,14]. The existing programs are mainly concentrated on first- and second-year MBO pupils, while less attention is paid to third- and fourth-year MBO pupils, partly due to the increasing difficulty of reaching them as a consequence of their internship commitments [14]. Moreover, the prevention programs are inadequately tuned to the influence of the direct social environment (that is, friends, peers and parents) that is related to the heavy drinking patterns of this specific target group [3]. Therefore, it is essential to develop and evaluate the effectiveness of alcohol prevention programs aimed at adolescents following the lower education levels.

Prior research has demonstrated that web-based brief alcohol interventions can be effective in reducing heavy alcohol use in adolescents and students [15-21]. Interventions delivered electronically via the internet have large practical advantages compared to the more conventional methods [22-24]. The internet is easily accessible and particularly appealing to young people. Furthermore, it allows the participants to access the intervention in the privacy of their homes at a convenient time, which may enhance their feelings of anonymity. Brief interventions are especially easy to implement by creating links on websites or providing the link of this website in promotion and education materials. Moreover, these interventions can be provided in an automated, cost-effective and flexible way [25]. Finally, the majority of adolescents in Western countries have access to the internet and make frequent use of internet technologies [26,27], which make web-based brief alcohol interventions particularly suitable for our target group.

### Objectives and hypotheses

The objective of the study described in this protocol is to evaluate the effectiveness of the What Do You Drink web-based brief alcohol intervention among heavy-drinking adolescents aged 15 to 20 years with a low educational background. A two-arm parallel-group randomized controlled trial will be conducted with two follow-up assessments (that is, after one and six months) to examine the effectiveness of the intervention. Two hypotheses will be tested. First, we expect that a larger percentage of participants in the experimental condition will drink within the normative limits of the Dutch National Health Council for low-risk drinking [28] when compared to the control condition as a result of the WDYD intervention. This means that participants’ consumption will not exceed a mean heavy alcohol use consumption of more than seven (girls aged 15 to 16 years), twelve (boys aged 15 to 16 years), fourteen (women aged 17 to 20 years) or twenty-one (men aged 17 to 20 years) glasses of standard units of alcohol per week and/or, in the case of binge drinking, five or more glasses of standard units of alcohol on one drinking occasion at least once per month and week for boys and girls aged 15 to 16 years and men and women aged 17 to 20 years, respectively, at one month and six months after the intervention. Second, we expect that participants in the experimental condition will reduce their mean weekly alcohol consumption and frequency of binge drinking. Thus, it is hypothesized that exposure to the WDYD intervention will be more effective compared to no intervention.

## Methods/design

### Trial design

The effectiveness of the What Do You Drink web-based brief alcohol intervention will be tested in a two-arm parallel-group cluster randomized controlled trial. Participants comprise approximately 750 heavy-drinking adolescents with a low educational background aged 15 to 20 years: 375 are in the experimental condition (web-based brief alcohol intervention) and 375 are in the control condition (no intervention).

### Procedure and participants

Participants will be recruited at VMBO and MBO institutions in the Netherlands. The VMBO and MBO institutions will be selected from a list of all educational institutions in different regions in the Netherlands. The selected educational institutions will receive an invitation letter with additional information about the study. A standardized cover story will be used in which institutions are informed that their students will participate in a study examining newly developed health education materials addressing alcohol use. After two weeks, the institutions will be contacted by telephone to establish whether or not they are willing to participate in the study. Those institutions that are willing to cooperate in the study will be asked to participate with as many as possible classes. Additionally, they will be requested to distribute letters to the parents of adolescents aged 15 to 16 years to inform them about the institution’s study participation. The parents will be given the opportunity to refuse participation by email or telephone during the entire study period. The informed consent materials will state clearly the expectations of frequency, duration and extent of study participation.

From the participating institutions, none of the adolescents will be excluded from study participation to avoid stigmatization and social exclusion. However, after the recruitment and enrolment of the institutions in the trial, an online baseline assessment will be carried out to establish whether the adolescents of the participating classes can be included in the study sample. Therefore, participants must: 1) be between 15 and 20 years old, 2) report heavy drinking in the past six months, and 3) be willing to change their alcohol consumption. Our definition of heavy drinking is based on measures of heavy alcohol use and binge drinking, which differs across participants’ sex and age. To fulfill the sample inclusion criteria, adolescents should be a heavy alcohol user and/or a binge drinker. However, problem drinkers who show symptoms of alcohol abuse or dependence (that is, an AUDIT score of 20 or above [29]) and/or of receiving treatment for alcohol-related problems will be excluded from participation since the WDYD intervention focuses on the prevention of heavy drinking rather than the prevention of problem drinking. The Ethical Committee (ECG) of the Faculty of Social Sciences of Radboud University Nijmegen in the Netherlands has approved the trial protocol.

### Intervention

Originally, the WDYD intervention had been developed for heavy-drinking young adults aged 18 to 24 years. Therefore, minor adaptations have been made concerning the usability (that is, use of language) of the intervention to make it more appropriate for the target group of adolescents with a low educational background aged 15 to 20 years. Detecting and reducing heavy drinking of adolescents who are willing to decrease their alcohol consumption is the main aim of the WDYD intervention. Motivational Interviewing principles [30] and parts of the I-Change model [31] are incorporated in the intervention, in which knowledge, social norms, and self-efficacy are embedded as the most changeable determinants of behavior change. To increase adolescents’ motivation to adapt their drinking behavior, discrepant personal information is presented [32]. Therefore, the first part of the WDYD intervention consists of a screening procedure and a form of personalized feedback based on the screening outcomes. The second part of the WDYD intervention focuses on goal-setting, action planning, and strengthening adolescents’ drinking refusal self-efficacy (for more details of the intervention see [33]).

### Intervention conditions

Participants in the experimental condition will be exposed to the WDYD intervention, while participants in the control condition will receive no further intervention.

### Data collection

**Figure 1:**
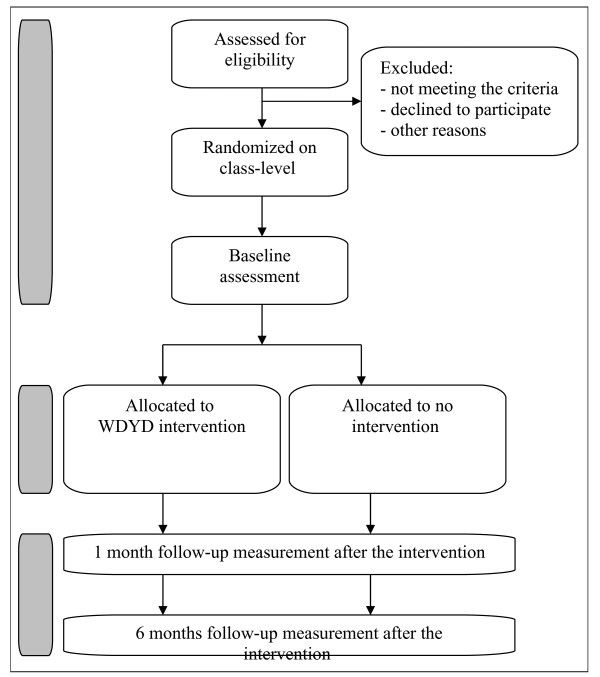
Study design.

The recruitment, enrolment in the trial, online baseline assessment, and randomization is scheduled during the period of October to December 2011. The follow-up assessments will be obtained one and six months after the intervention, that is, in the period November to December 2011 and April to May 2012. In addition, the participating VMBO and MBO institutions will be offered an incentive in the format of the DVD workshop ‘Advertisement agency’, after their students have completed the total follow-up period. The workshop, designed by the Trimbos Institute (Netherlands Institute of Mental Health and Addiction), is developed for adolescents and focuses on alcohol, tobacco, and drugs use and peer pressure. An overview of the measurements is given in Figure 1.

### Outcomes

Primary outcomes measures are: 1) the percentage of participants who drink within the normative limits of the Dutch National Health Council for low-risk drinking, 2) reductions in mean weekly alcohol consumption, and 3) frequency of binge drinking. The secondary outcome measures include the alcohol-related cognitions, attitudes, self-efficacy, and subjective norms.

The Dutch version of the Alcohol Weekly Recall [34] will be used to measure participants’ average glasses of standard alcohol units in the previous week. To assess frequency of binge drinking, respondents will be asked how often they consumed five or more glasses of standard alcohol units on one drinking occasion at least once per month (boys and girls aged 15 to 16 years) and week (men and women aged 17 to 20 years) in the past month and week, respectively.

Attitudes towards alcohol use reflect the extent to which respondents have a positive or negative regard of alcohol use. Respondents will be asked about the perceived effects of alcohol (for example, ‘Drinking alcohol makes me feel less shy’ and ‘Drinking alcohol makes me fat’). Respondents will complete 10 positive and 10 negative attitude items and respond on a 4-point scale ranging from (1) ‘strongly disagree’ to (4) ‘strongly agree’.

Self-efficacy will be measured with a modified version of Young’s Drinking Refusal Self-Efficacy Questionnaire Revised Adolescents Version (DRSEQ-RA: [35,36]), which assesses respondents’ ability to resist drinking alcohol in various situations. This measure was modified by adding additional risk situations in which respondents find it hard to resist alcohol. Additional items were generated by the authors (for example, ‘When I have finished my exams’). Respondents will complete 26 items and respond on a 6-point scale from (1) ‘I am very sure I cannot resist alcohol’ to (6) ‘I am very sure I can resist alcohol’, with higher scores reflecting higher DRSEQ-RA. The measure incorporates three subscales that reflect drinking refusal self-efficacy relating to social pressure (for example, ‘When my friends are drinking’), emotional relief (for example, ‘When I am angry’) and opportunity to drink (for example, ‘When I am watching TV’).

Descriptive norms will be assessed by measuring perceived alcohol use among best friend, partner, parents, and typical same-sex student. Respondents will be asked about the frequency of their best friend’s/partner’s/parents’ and typical same-sex student’s alcohol use in the previous four weeks. The respondents can respond on a 6-point scale ranging from (1) ‘have not been drinking’ to (6) ‘every day’ [37]. The intensity of their best friend’s/partner’s/parents’/typical same-sex student’s drinking will be assessed by asking the respondents the number of glasses of standard alcohol units their best friend/partner/parents/typical same-sex student had drunk in the previous week in the contexts of at home and outside the home [38]. By asking about these four specific situations, respondents are forced to increase the reliability of response [39]. The scores on these four questions will be summed up to get an indication about the total number of glasses of standard alcohol units the best friends/partner/parents/typical same-sex student of each adolescent consumed in the past week.

Injunctive norms will be assessed by measuring the perceived acceptability of drinking among adolescent’s best friend, partner and typical same-sex student. Respondents will be asked: ‘Do you think that (1) your best friend, (2) your partner and (3) the typical same-sex student would mind if you drink a lot?’ Responses will be coded using a 4-point scale anchored by (1) ‘not at all’ to (4) ‘a lot’. A higher score indicates more liberal norms towards adolescent drinking. Thus, both proximal and distal reference groups will be used to assess social norms.

### Sample size

The power calculation of our study reflects the notion that we aim to detect an increase in the percentage of participants showing low-risk drinking after one month of 42% in the experimental group versus 31% in the control group [40]. A total sample size of 750 respondents (n = 375 per condition) will be required to test the hypothesis in a two-sided test at alpha = 0.05, a power of (1-beta) = 0.80, and expecting a worst-case scenario of totally 15% loss-to-follow-up after randomization. The fact that the data are clustered (participants are nested in classes) was taken into account in the power calculation. The intraclass correlation is expected to be between 0.03 and 0.06 indicating that there is a low degree of similarity between participants within classes [41].

### Randomization

Randomization will occur by class level within the educational institutions to avoid contamination between the conditions. Thus, classes of adolescents from a VMBO or MBO institution will be randomly assigned to either the experimental or the control condition. A blocked randomization scheme (block size four) will be used. An independent researcher of the Behavioural Science Institute will perform the allocation with a computerized random number generator after baseline assessment.

### Statistical methods

Descriptive analyses will be conducted to explore whether the randomization has resulted in a balanced distribution of participants’ demographic characteristics across conditions. The potential non-independence of the clustered data, due to the fact that participants are nested in classes, will be taken into account in the analyses.

Data will be analyzed in accordance with the intent-to-treat principle and the completers-only framework in SPSS and/or Mplus. For the intention-to-treat analyses, missing data at follow-up assessments will be handled using multiple imputations using the predictive mean matching method (continuous data) and the logistic regression method (categorical data). Additionally, completers-only analyses will be conducted on participants with scores on all measurements.

Logistic and linear regressions will be performed in both the intention-to-treat and the completers-only analyses to test how the WDYD intervention is related to the alcohol outcomes (that is, heavy drinking, mean weekly alcohol consumption, and frequency of binge drinking) one and six months after the intervention. Besides testing the main effects of the WDYD intervention, moderating effects of age, sex, and drinking status will be investigated to establish whether subgroups are more likely to benefit from the WDYD intervention. Moreover, mediating processes will be examined to: 1) test whether the WDYD intervention modifies the mediating factors (that is, attitudes, social norms and self-efficacy: ASE-model [42]), 2) provide insights into how the WDYD intervention achieves its effects (that is, which mediating factors are modified by the WDYD intervention that are related to alcohol outcomes), and 3) reveal which mediating factors are the most important for realizing change in the alcohol outcomes [43]. Three steps will be performed to analyze the mediating effects [44]. First, it will be analyzed whether the WDYD intervention has an effect on the mediating factors. Then, the effects of the mediating factors on the alcohol outcomes will be analyzed, while controlling for the effect of the WDYD intervention. Finally, it will be analyzed whether or not the size of the mediated effects are statistically significant [44,45].

The study will be performed in accordance with the CONSORT (Consolidated Standards of Reporting Trials) guidelines [46].

## Discussion

The current study has described a study protocol for evaluating the effectiveness of the What Do You Drink web-based brief alcohol intervention for 15- to 20-year-old adolescents with a low educational background by using a two-arm parallel-group cluster randomized controlled trial. Evaluation of the WDYD intervention will provide insights into its effectiveness, which will be communicated to scientists and health professionals.

One of the strengths of this program concerns the theoretical underpinning of the WDYD intervention, which is based on Motivational Interviewing principles and social influence models. Both have been proven to be effective when used in web-based brief alcohol interventions aimed at reducing heavy drinking among students [15-19,21]. Further, the web-based approach of the tailored intervention may be more effective over the more traditional delivery methods [32]. In addition, WDYD is a short intervention (about 20 minutes), which makes it less time-consuming than regular prevention programs and, therefore, easier to implement. Finally, standardized responses will be ensured by providing an overview of standard units for various beverages. However, this study has several limitations that are worth mentioning. First, a convenience sampling strategy will be used to recruit participants at VMBO and MBO institutions, which may limit generalizability. Second, all measurements are based on self-report measures, possibly resulting in over- or underreporting of alcohol outcomes due to social desirability [47]. However, confidentially will be assured in the informed consent, which make the self-report measures of alcohol use reliable and valid [48,49]. Third, despite clustering the randomization at class level, participants in the control condition could have been exposed to the WDYD intervention when they have friends in the experimental condition who have shared the link of the intervention. However, contamination between conditions is expected to be minimal since the WDYD intervention is neither yet available to the general public nor online. Moreover, contamination between conditions will be evaluated by asking all participants if they have actually seen the WDYD intervention by presenting screening shots of the homepage of the intervention to reduce participants’ memory bias. Fourth, it may be the case that being exposed to the intervention or control condition influences the way participants perceive the alcohol use and acceptability of drinking of the reference group under investigation. However, adolescents are rather accurate in estimating their best friend’s drinking behavior [50]. Finally, the development of the effects of the intervention over time cannot be examined, since there are only two follow-up assessments at one and six months after the intervention.

### Trial status

The status of the trial is ongoing at the time of manuscript submission. The recruitment of participants is expected to be completed by December 2012.

## Competing interests

All authors declare that they have no competing interests.

## Authors’ contributions

CV is responsible for the data collection, data analysis, and reporting the study results. The other authors are supervisors and grant applicators. All authors read and approved the final protocol.
